# Editorial: Data science and machine learning for psychological research

**DOI:** 10.3389/fpsyg.2025.1634692

**Published:** 2025-06-16

**Authors:** Chong Ho Yu

**Affiliations:** Department of Mathematics, Hawaii Pacific University, Honolulu, HI, United States

**Keywords:** data science, machine learning, artificial intelligence, data mining and knowledge discovery, classical statistics

The landscape of psychological and social science research is undergoing a profound transformation, and the catalyst is clear: the integration of data science and machine learning methodologies. As evidenced by these compelling studies in the Research Topic “*Data science and machine learning for psychological research*,” the ways researchers collect, process, analyze, and interpret data are being redefined, ushering in an era that demands new skills, mindsets, and scientific philosophies.

The paper ‘‘*Diurnal patterns in Twitter sentiment in Italy and United Kingdom are correlated*,” authored by Wang S. et al. investigates whether emotional patterns on Twitter exhibit consistent circadian rhythms across different cultural settings during the 2020 COVID-19 lockdowns. They collected over 2 million tweets from 9 Italian cities and nearly 34 million tweets from 54 UK cities, sampled hourly. To analyze emotional expression, they used LIWC2015 psychometric tools, translating Italian tweets into English via machine translation (natural language processing) to maintain comparability. The methodology strongly leverages data science techniques, including time series analysis, Fourier transforms, and statistical modeling (such as average daily/weekly profile construction and variance analysis).

The article “*The effect of reading engagement on scientific literacy—an analysis based on the XGBoost method*” by Cao et al. explores how students' engagement in reading influences their scientific literacy, using machine learning techniques. Drawing on data from the 2018 PISA assessment in China (specifically Beijing, Shanghai, Jiangsu, and Zhejiang), the authors selected 36 variables encompassing background information and different dimensions of reading engagement (behavioral, affective, and cognitive) to predict scientific literacy scores. They applied the XGBoost algorithm, a cutting-edge machine learning method known for handling complex, large-scale data, along with SHAP (Shapley Additive Explanations) to interpret the model globally and locally. The research study confirms that machine learning models like XGBoost, combined with SHAP for interpretability, offer robust, flexible alternatives to traditional statistical methods in educational assessments. This study not only emphasizes the role of high-level cognitive reading skills in science learning, but also showcases the practical application of advanced data science techniques to educational research.

The article “*Psychological factors enhanced heterogeneous learning interactive graph knowledge tracing for understanding the learning process'*' by Wang Z. et al. proposes a new model, Psy-KT, to advance the field of educational technology by incorporating students' psychological states into knowledge tracing. Traditional knowledge tracing models mainly focus on historical exercise data and skill mastery but often ignore learners' mental states like frustration, confusion, concentration, and boredom. Psy-KT addresses this gap by building a heterogeneous learning interactive graph that captures interactions among students, exercises, and skills, and integrating psychological factors and a forgetting mechanism to simulate real-world learning dynamics. The methodology heavily utilizes data science and machine learning approaches, including Graph Neural Networks (GNNs), Gated Graph Neural Networks (GGNNs), attention mechanisms, and Item Response Theory (IRT) models, to predict students' future performance with high interpretability.

The article “*Predicting implementation of response to intervention in math using elastic net logistic regression*” by Wang Q. et al. investigates key predictors influencing U.S. elementary schools' adoption of math Response to Intervention (RTI) programs. Using data from the Early Childhood Longitudinal Study (ECLS-K: 2011), the authors employ advanced data science and machine learning methods, specifically random forest algorithms for missing data imputation and elastic net logistic regression with nested cross-validation for predictive modeling. They built 10 imputed datasets and tested four variable selection methods to create and validate robust predictive models. The final model achieved a high balanced accuracy of 0.852, demonstrating strong predictive capability.

The article “*Leveraging on large language model to classify sentences: a case study applying STAGES scoring methodology for sentence completion test on ego development*,” authored by Bronlet, explores the potential of using large language models (LLMs) to automate the classification of ego development stages, traditionally performed by human experts. Focusing on the STAGES scoring methodology, the study applies LLMs like GPT-4o and others to classify sentences based on cognitive maturity dimensions such as object awareness, individuality vs. collectivity, and cognitive orientation. While acknowledging some limitations in single-sentence precision, the study highlights the promise of LLMs for scalable, cost-effective psychological assessments and suggests future work incorporating even larger datasets and continuous expert feedback to improve model alignment.

Taking all of the above accomplishments into consideration, it is clear that today researchers are no longer confined to structured survey responses and small, manageable datasets. Data science techniques enable us to leverage both structured and unstructured data, such as free-form text from social media platforms or sentence completion tests in ego development studies. Natural language processing (NLP) technologies, including the use of large language models (LLMs), have transformed unstructured text into analyzable quantitative formats, a feat that was once inconceivable.

Sample size, once a limiting factor constrained by manual data collection methods, has exploded into the domain of “big data.” Studies examining millions of tweets or vast educational assessment databases demonstrate that large-scale, population-level insights are now within reach. These massive datasets, however, would be unmanageable without the sophisticated restructuring and automation tools that machine learning provides.

Analytics have evolved equally dramatically. Where classical statistical tools like hypothesis testing and *p*-values once reigned supreme, today's research increasingly employs algorithms such as random forests for imputation, XGBoost for predictive modeling, elastic net logistic regression for variable selection, and LLMs for nuanced text classification and scoring. These methods offer robustness, scalability, and predictive accuracy that traditional techniques cannot match.

It would be incorrect to declare classical statistics obsolete; there remains a place for *p*-values and hypothesis tests, particularly for foundational validation. However, the studies here clearly illustrate that psychology and the broader social sciences have entered a paradigm shift. Data science and machine learning are no longer optional complements; they are essential pillars of contemporary research methodology.

As researchers, we are standing at a crossroads. Embracing these advancements is not just an opportunity; it is a necessity for those who seek to push the boundaries of knowledge, to derive more actionable insights, and to remain relevant in an increasingly complex and data-driven world. Psychological and social science research must continue to evolve, and today's innovations are charting the course forward.

We call on researchers across disciplines to invest in these technologies, collaborate with data scientists, and innovate boldly. The future of our fields depends on it.

